# Process Mining Dashboard in Operating Rooms: Analysis of Staff Expectations with Analytic Hierarchy Process

**DOI:** 10.3390/ijerph16020199

**Published:** 2019-01-11

**Authors:** Antonio Martinez-Millana, Aroa Lizondo, Roberto Gatta, Salvador Vera, Vicente Traver Salcedo, Carlos Fernandez-Llatas

**Affiliations:** 1 Instituto Universitario de Tecnologías de la Información y Comunicaciones, Universitat Politècnica de València, Camino de Vera S/N, 46022 Valencia, Spain; arligar@upv.es (A.L.); vtraver@itaca.upv.es (V.T.S.); carferll@itaca.upv.es (C.F.-L.); 2 Dipartimento di Diagnostica per Immagini, Radioterapia Oncologica ed Ematologia, Università Cattolica del Sacro Cuore, 00168 Rome, Italy; roberto.gatta.bs@gmail.com; 3 MYSPHERA SL, Ronda Auguste y Louis Lumiere 23, Nave 13, Parque Tecnólogico, 46980 Paterna, Spain; svera@mysphera.com; 4 Unidad Mixta de Reingeniería de Procesos Sociosanitarios, Instituto de Investigación Sanitaria del Hospital Universitario y Politecnico La Fe Bulevar Sur S/N, 46026 Valencia, Spain

**Keywords:** process mining, analytic hierarchy process, operating rooms, usability, software, co-design

## Abstract

The widespread adoption of real-time location systems is boosting the development of software applications to track persons and assets in hospitals. Among the vast amount of applications, real-time location systems in operating rooms have the advantage of grounding advanced data analysis techniques to improve surgical processes, such as process mining. However, such applications still find entrance barriers in the clinical context. In this paper, we aim to evaluate the preferred features of a process mining-based dashboard deployed in the operating rooms of a hospital equipped with a real-time location system. The dashboard allows to discover and enhance flows of patients based on the location data of patients undergoing an intervention. Analytic hierarchy process was applied to quantify the prioritization of the dashboard features (filtering data, enhancement, node selection, statistics, etc.), distinguishing the priorities that each of the different roles in the operating room service assigned to each feature. The staff in the operating rooms (*n* = 10) was classified into three groups: Technical, clinical, and managerial staff according to their responsibilities. Results showed different weights for the features in the process mining dashboard for each group, suggesting that a flexible process mining dashboard is needed to boost its potential in the management of clinical interventions in operating rooms. This paper is an extension of a communication presented in the Process-Oriented Data Science for Health Workshop in the Business Process Management Conference 2018.

## 1. Introduction

Operating rooms (ORs) are an essential and central element in modern hospitals [1]. Cost estimations of ORs are around 16% and 20% of total hospital budget, due to the amount and heterogeneity of human resources involved and the high technology used [2,3]. Efficiency in the management and interventions in ORs aims to reduce the non-occupation time of the surgical blocks and the way of organizing the different types of interventions to optimize medical teams (surgeons and nurses). To achieve a good efficient rate in ORs, it is crucial to have a protocol and a highly skilled staff to take over daily decision making for scheduled and unscheduled interventions [3].

Recent reviews have concluded that managerial surgeons make decisions to increase the clinical work per time-unit in individual ORs and that command displays may be an effective way to gain efficiency [4]. This behavior is well-known as reactive scheduling, which involves schedule definition and posterior assessment [5]. Schedule definition entails foreseeing the starting, duration, and ending times for the regular operation sequences (preparation, anesthesia induction, intervention, wake-up, and turnover) in terms of time and resources. Posterior assessment monitors the schedule execution and adapts the planned schedule to deal with unexpected events [6]. A reactive scheduling process occurs when unexpected events or disruptions occur along the process [7]. Nevertheless, the main disadvantage in OR planning and management is that processes are often recorded manually by nurses. This issue has already been identified as a bottleneck in the performance assessment of ORs [8]. Moreover, manual notes can also lead to bias: Unnecessary delays, underuse of the operation rooms, unnecessary transfers, etc. In addition, it could cause an increase in the probability of the adverse effects in the surgical process, which, according to the authors of [9], stand for the 40% of all the adverse effects in hospitals.

Information and communications technology (ICT) can provide tools and systems to support both the programming and assessment of operations. To perform this tracking process, a pragmatic task Gonzalez et al. proposed a semi-automatic information collector [10]. Nowadays, some hospitals are equipping themselves with real-time location systems (RTLS) to manage the location of patients and assets that could help to optimize the management of ORs by applying process mining (PM) techniques.

An example of the use of the extracted data from the RTL systems to manage patient locations is introduced by Reference Fernandez-Llatas et al. [11], in which PM is applied to perform an analysis of operation sequences and locations in the ORs, showing the most common paths and insights of the entire process in ORs based on RTLS data. In this study, researchers developed a front-end application to analyze OR processes, providing a complete suite of tools to discover, compare, and enhance surgical processes.

Technologies should be presented in a meaningful way to the OR staff to ensure a successful deployment. The application of computer decision systems in an interactive way will not only increase their effectiveness and efficacy [12], but also involve the staff of ORs in the process of knowledge extraction, avoiding frustrations using technology for managing complex processes [13].

Process mining has multiple unknowns when landing to real applications when the intended user is the clinical staff. In this paper, we report a study based on the analytic hierarchy process (AHP) to identify the preferred features of a web-based process mining dashboard. This front-end has been presented in Reference [11]. To distinguish the preferences of each role, we grouped the users in three groups: Manager, hospital staff, and technical staff of the ORs. We obtained feature prioritization of 10 subjects, including the three roles. The AHP is particularly effective for quantifying experts’ opinions that are based on personal experience and knowledge to design a consistent framework for the application of process mining in ORs.

This paper is an extension of the communication entitled “Expectations from a Process Mining Dashboard in Operating Rooms with Analytic Hierarchy Process”, presented in the Process-Oriented Data Science for Health Workshop in the Business Process Management Conference celebrated in Sidney (Australia) in September 2018 [14].

## 2. Related Work

Real-time location systems (RTLS) monitor the position of a moving element with a given sampling frequency. In a hospital, moving elements are equipped with an active or passive element (tag), which is identified when it is nearby a beacon. In our study, patients were the moving elements who wore a wristband with the tag before entering the operating room service.

Lean principles present a condensed primer of RTLS in a health care environment [15]. Throughput is a key performance indicator for a patient pathway across a facility, which, linked to RTLS, could provide valuable information, such as waiting times and resource utilization [16].

RTLS systems in 23 hospitals in the US were analyzed from a qualitative perspective in Reference [17]. In this work, researchers observed the systems in use and conducted 80 semistructured interviews with hospital personnel and vendors. Authors found asset tracking the best feature and identified several obstacles related to the technical set-up and organizational context. According to the authors, RTLS must go beyond the simple deployment of technology to be effective.

**Figure 1:**
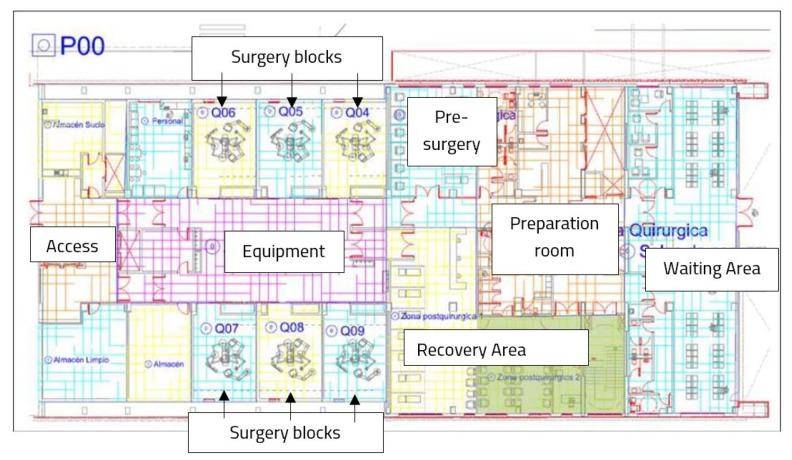
Composition of the operating room service.

Specifically, an OR service consists of several spaces, each of them equipped with a beacon to identify a patient whenever he/she goes to that specific area (Figure 1). The regular flow of a patient starts in the preparation area, continues in the pre-surgery room or in the operating room, and ends in the recovery area.

**Figure 2:**
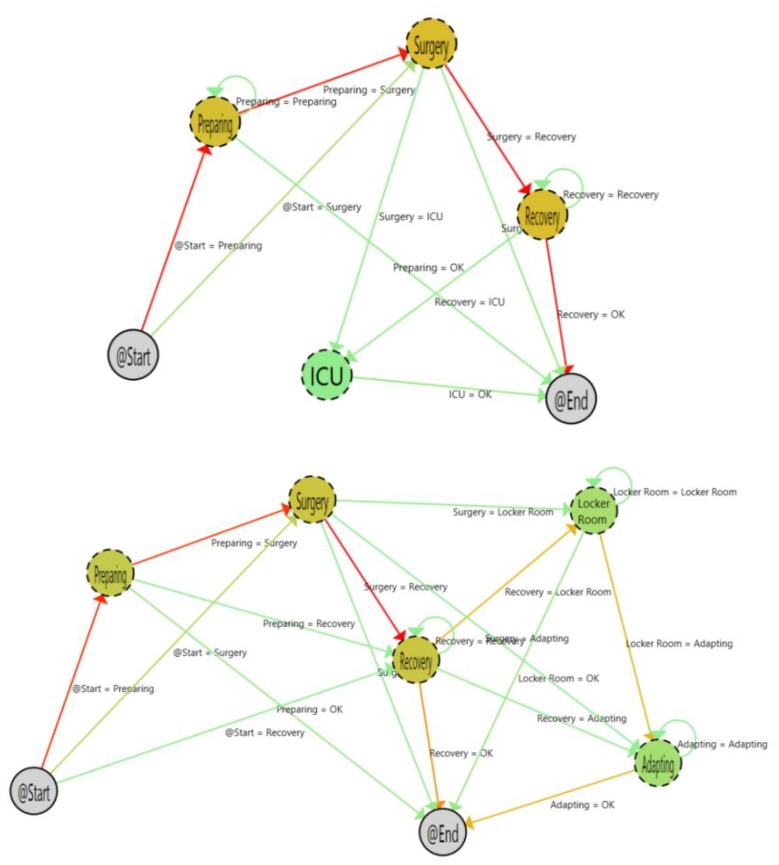
Example of the inferred and enhanced work flows of patients across the operating room service [11].

The application of process mining techniques in combination with RTL systems provided an easy-to-use and unobtrusive way to achieve a view of the deployed process and assess the time that patients, professionals, and assets are in each stage or location. In this paper, we analyze the web-based dashboard to perform process mining discovery and enhancement analytics, which has already been done by Fernandez-Llatas et al. [11] (Figure 2).

Analyzing RTLS data from a discovery perspective and enhancing these work flows with information related to the average time and overload, it is possible to create pre-programmed contingency plans for the management and allocation of resources of the operating rooms. However, why are these promising applications still not widely used in the clinical context? Instead of using a semistructured interview, we used the analytic hierarchy process [18] to quantify the features of a dashboard for discovering and enhancing processes based on RTLS data in an OR service.

## 3. Materials and Methods

It is necessary to identify the characteristics of the dashboards and relate them with the preferences and how and why those could impact clinical use.

The classic managerial structure of a hospital consists of a compartmental service-oriented organization [19]. This structure does not consider the flow of patients between services and how the decisions in one service could affect the following from the perspective of a process. Process-based management is focused on an organizational understanding of the medical and administrative processes in a hospital by collecting and analyzing collaborative information from professionals and their organizational units.

The dashboard analyzed in this study provides functionalities to aggregate data from an RTLS in an OR and analyze the flow of patients across the process of a surgical intervention. The dashboard allows to discover and enhance the patients’ trajectories and the time they are at each stage and extract insights from processes. The information obtained from the dashboard is based on trustworthy data automatically collected by the RTLS and, thus, the information extracted depicts the actual performance and deployment of the processes in the OR. RTLS data are enriched with metadata of the clinical intervention (e.g., type of surgery). Our aim is to discover which of the characteristics of the dashboard are perceived as useful by OR staff to help them with their daily clinical and administrative work. In this section, we describe the characteristics of the process mining dashboard (Section 3.1), the methodology applied to perform the feature prioritization by the OR staff (Section 3.2), the questionnaires and tools used to do the feature prioritization (Section 3.3 and Section 3.4), and the characteristics of the participants in the study, composed of the full team of ORs in Hospital General of Valencia (Section 3.5).

### 3.1. Analyzing Operating Rooms Processes with PALIA

**Figure 3:**
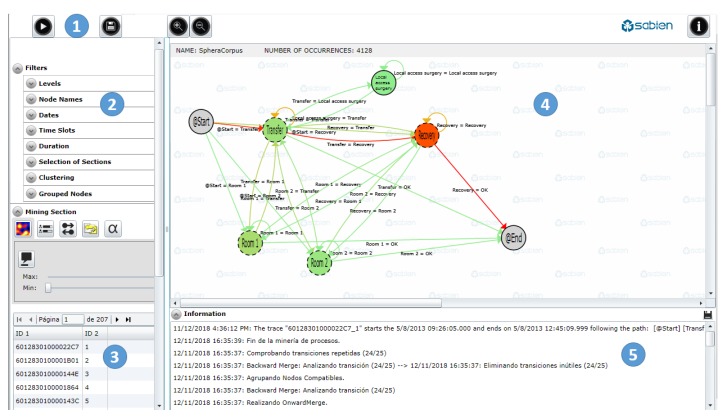
Areas of the process mining dashboard.

PALIA consists of a web-based dashboard, which allows to perform process mining analysis on a given dataset. The software (Figure 3) is composed of three major areas: Filters, for the selection of the data; mining, for the configuration of the visualization of work flows; and information, to show the information about the operation tool and the selected tracks. These three major areas are divided into five functional areas:

1. **Filters (1–2)**, for accessing basic functionalities of the dashboard, such as the selection of the input data. There are several types of filters depending of the type of data and the required information: Dates, times, durations, type of intervention, etc. This component shows the percentages of the samples meeting the filtering characteristics, so the user can have an idea of the extension of the selected subgroup of data.
2. **Miner (3–4)**, for the configuration of the work flow visualization. Graphical representations of the inferred processes are depicted in the central part of the web-tool (4) by means of OR states (nodes) and transitions (arrows). The visualization component allows to add meta-information to the work flow, for instance, rendering heat maps to discover frequencies or occupation in the ORs. There is also a list with all the samples selected with the filters (1–2).
3. **Information (5)**, which shows details about how the process mining algorithm was applied and also features of the selected samples: Information on the number of merged branches, a log on how the process mining algorithm analyzed the events and infers work-flows, errors on data selection, and statistics on the transitions and states in the work-flow.

PALIA works in the following way: First, a comma-separated value file containing the RTLS data is loaded using a default file dialog window. Then, the user is able to filter the data and apply a discovery topological algorithm. The inferred work-flow appears in the screen with nodes and arrows, which represent the track followed by the patients across the surgical process (Preparation—Surgery—Recovery—Intensive care unit—Locker room—Adaptation).

### 3.2. Analytic Hierarchy Process to Determine Priorities

AHP is a methodology for decision-making which aims at solving complex problems [20]. It allows to quantify opinions and transforms them into a coherent decision model. The process is derived from a pairwise comparison using a numerical scale. AHP has found its widest applications in multifactor decision-making, planning, and resource allocation, and in conflict resolution [21,22]. AHP is a method which incorporates benefits and risks explicitly by combining the importance of differences in probabilities of outcomes related to alternatives and the weighting of the importance of those outcomes [23]. Unlike other methods for feature selection, such as conjoint analysis [24], AHP is specially concerned with the consistency of choices and their measurement and dependencies between the groups of elements [25]. Some key and basic steps of AHP were introduced by Pecchia et al. [18]:

- Define the problem.
- Broaden the objectives of the problem or consider all actors, objectives, and its outcome.
- Identify the criteria that influence the behavior.
- Structure the problem in a hierarchy of different levels constituting the goal, criteria, subcriteria and alternatives.

Our aim was to develop a hierarchy of elements grouped into categories to describe the functionalities of the PM tool used to manage OR processes. These categories were ranked using questionnaires to extract the relative importance of each need per category (local weights, LW), the relative importance of each category (category weights, CW), and the importance of each need compared to all the others (global weights, GW) [26].

### 3.3. Applying AHP to PALIA

AHP was applied to this study because of its inherent capability of prioritizing characteristics in a qualitative and quantitative way. The application of AHP involves six factors [21]: The hospital was motivated to adopt PALIA to support OR process management and was committed to implementing the decision and involved staff from the OR department. Stakeholders were active participants in the entire decision process from development to implementation. In order to identify the elements and the categories of the hierarchy, we used the different functionalities of PALIA. The hierarchy is composed of four levels, which have a 1:*n* relationship with the three functional areas described in Section 3.1. The first hierarchy level is generic and only describes the functional area (filters, miner, and log). The second hierarchy level describes the main features of the functional area (for filters, it contains dates, times, duration, type of intervention, type of OR, etc.). The third hierarchy level contains details of the main features within the functional area (in filters, for the type of intervention, it contains details of the medical service, the type of program, the surgical process, the surgeon in chief, etc.). The fourth and final hierarchy level contains low granularity details. To create the hierarchy tree and collect the answers, we used the web application BPMSG AHP On line System (https://bpmsg.com/academic/ahp.php). In this study, we focused on two hierarchy priority levels:

1. **Hierarchy Level 1**, functional Area: Filter, miner, and information.
2. **Hierarchy Level 2**, features of the functional level (Table 1, Table 2 and Table 3).

### 3.4. Qualitative Study

In addition to quantitative prioritization and selection of the features in the dashboard with AHP, the study included an interview using a usability questionnaire implemented on the basis of the system usability scale (SUS) methodology. SUS is a balanced questionnaire comprising 10 alternating positive and negative statements [27], which is robust against extreme response bias [28]. To gain more insights about the dashboard usability, we adapted SUS to include seven closed items (to be rated in a 5-point Likert scale) and three open questions. The questionnaire is described in Table 4.

### 3.5. Participants

Table 5 shows the profiles of the involved participants and their specific role into the OR service. The project identification code is 812386, under European Union’s Horizon 2020 research and innovation program. Only one of the initial 11 participants (complete OR staff) was unable to fill the AHP questionnaire successfully and was discarded for the analysis. The respondents, who signed the informed consent of this study, were employers of the *Hospital General de Valencia*, one of the four hospitals of reference at the city, covering a population of 350,000 inhabitants. It has 27 operating rooms and in 2014, it registered 26,497 surgeries [29].

The study was organized in a session lasting 3 h. First, the dashboard was introduced to the study participants in the Hospital General premises. The first part consisted of a walkthrough of the application, in which we analyzed a simple dataset of one-day RTLS data with two types of surgical interventions. The second part consisted of analyzing a complete dataset containing three months of RTLS data with multiple surgical interventions. At this point, participants were familiar with the tool and were ready to prioritize features. The third and final part consisted of the fulfillment of questionnaires for the quantitative and qualitative study.

## 4. Results

A total of 10 questionnaires were collected after the session held in the University General Hospital of Valencia with professionals working in the OR service. For the analysis of the responses, participants were grouped into three categories depending of their roles within the OR service. The roles were defined according to the type of tasks and responsibilities each professional had in the OR service, so each participant was assigned to a unique category. The category of managers was composed of professionals with a high-level organization profile, who are responsible for the general performance of the service, human resource management, and reporting to the board of the hospital. The category of clinical staff was composed of three general surgeons and three nurses. The Technical category was composed of engineers who are responsible of the information technology systems in the ORs.

The AHP questionnaire allows to assign priorities for each of the features contained in the defined hierarchy levels, each of which correspond to a particular functional area of the PALIA web-tool.

The overall analysis of priorities shows a 57% level of consensus (relative homogeneity β = 77%), in which the miner component achieves the higher priority rates for the features of visualization (heat maps) and views selection (maximum occupation and current occupation). Collecting the relative priorities within each of the three main categories, we could extract the importance that each group of users assign to each of the PALIA functional areas.

**Figure 4:**
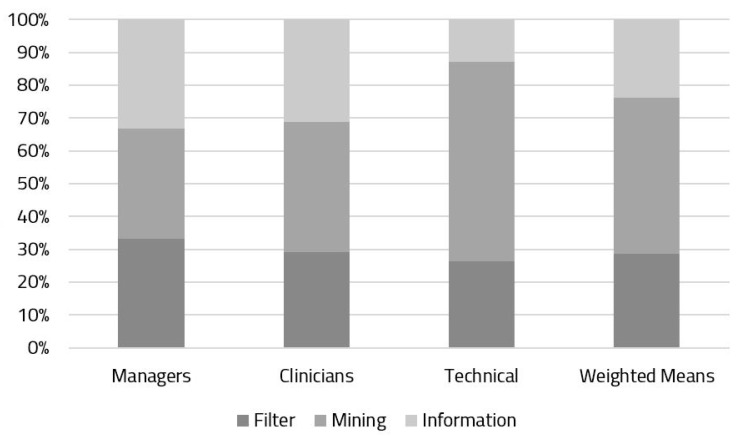
Assigned priorities for the functional areas.

Figure 4 depicts the relative weights (%) assigned to each of the modules. The manager and clinical user groups showed a similar consensus on the prioritization of functional areas, whereas the group composed of the technical staff provided more priority to the mining component. The stack in the right part of Figure 4 is a weighted mean of the relative priority assigned by participants, in which we can see that the mining component is still the most important, and the two other components (filters and information) share a similar, lower importance.

**Figure 5:**
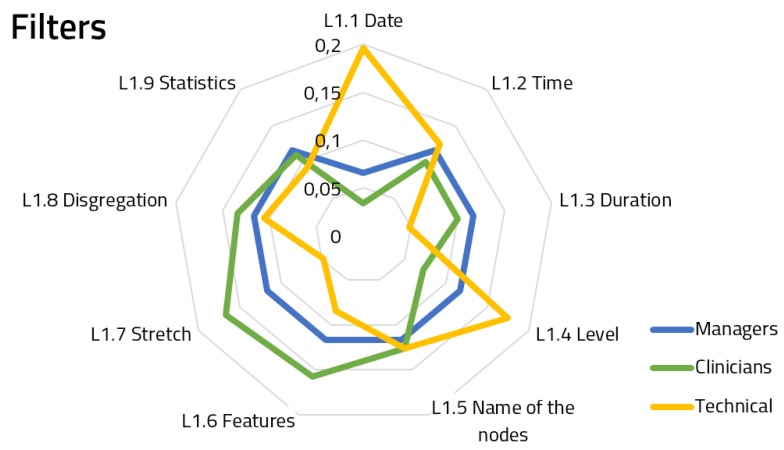
Priorities for filter functional module features.

**Figure 6:**
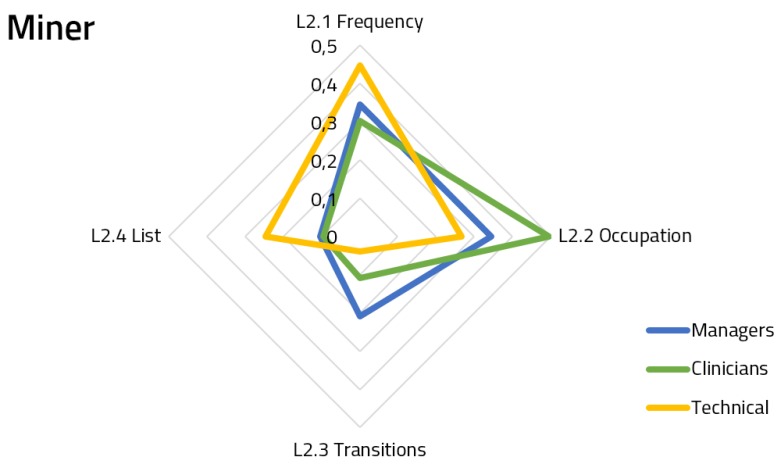
Priorities for miner functional module features.

**Figure 7:**
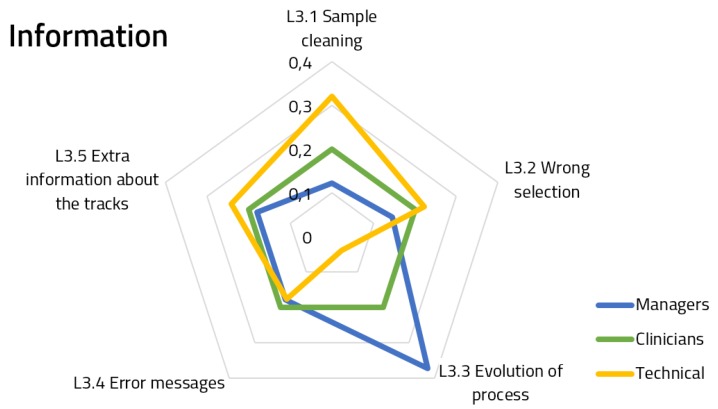
Priorities for information functional module features.

Figure 5, Figure 6 and Figure 7 show the spiderweb diagram containing the assigned priorities within Hierarchy Level 2 for each of the features, splitting responses by user groups.

Regarding the filters (Figure 5), we can see a similar consensus between the managers and the clinicians, whereas the technical staff is weighting two features which were not that relevant for the former groups: Date and level selection. The answers for the filter component achieved a 65.7% group consensus.

Regarding the miner (Figure 6), which was the most weighted component overall, we can see that clinicians were more interested in knowing the occupation of the rooms flow, but with respect to the frequency, there are similar priorities between them and managers. This figure also shows that only the technical staff is interested in having the list of samples which comprised the work-flow. The answers for the miner component achieved a 78.5% group consensus.

Regarding information (Figure 7), we can see that the priorities assigned by the clinicians are the same for each feature, which could indicate a strong consensus or that the features are not relevant to this group. For the other two groups, there are two different features which received a significant different prioritization. Managers are more likely to have information about how the process evolved over time, and technical staff is more prone to having information about the sample cleaning (which, moreover, has a similar weight to other related features, such as the wrong selection of samples and extra information).The answers for the Information component achieved an 80.8% group consensus.

**Figure 8:**
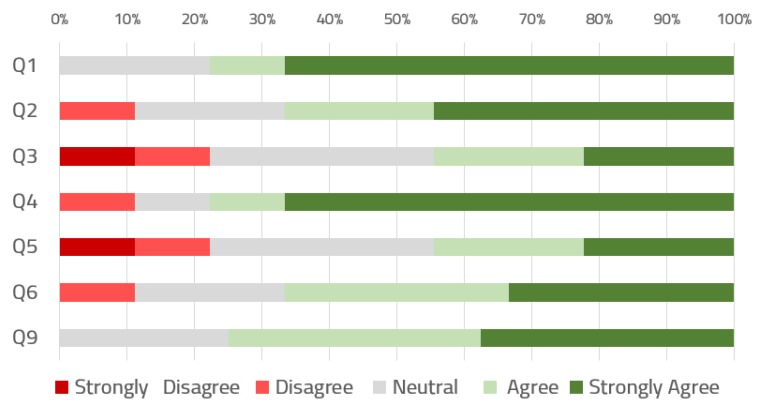
Priorities for information functional module features.

Figure 8 draws the distribution of the results obtained from the questionnaire (only for the closed questions with the 5-item Likert scale, as shown in Table 4). Sixty-seven percent of the users would like to use the dashboard on a daily basis to organize and supervise ORs, and the same percentage think they could use the dashboard without the support of a technical person. Between 44% and 33% of the respondents indicated a strong agreement to the questions related to simplicity, consistency, and intuitiveness. Only two questions had a 10% of strong disagreement and 22% of strong agreement, and these were related to the ease of use and integration of the functionalities.

Regarding to the open-ended questions, respondents highlighted the need to be able to identify patients through their number of clinical history instead of by tags ID, as it is now (from the RTLS). In addition, several of them also highlighted the potential usefulness of being able to introduce contextual factors and information related to incidences or intervention cancellations. One manager also pointed out the suitability of this framework in the management of bed occupancy.

## 5. Discussion

In this paper, we evaluated a dashboard for executing process mining analytics involving end users working in the OR service of a reference hospital. The dashboard used data from RTLS to discover, enhance and assess work flows of patients. The evaluation was two-fold, using first the AHP for prioritizing features in the dashboard and, subsequently, a SUS-based questionnaire.

AHP questionnaires allowed us to extract valuable and quantifiable information about the use of a dashboard for the exploitation of process mining on RTLS samples in the ORs of a hospital. The sample size of the questionnaire could be considered small (*n* = 10) to provide statistically significant findings. Nevertheless, this population contains all professional staff working in the operating room services in the hospital, and our results should be considered as a starting point to perform large-scale evaluations.

The assessment of PALIA features allow to enhance the communication with the clinical environment to create a powerful and usable tool for the application of process discovery on RTLS data. The distinction between the different groups according to their roles allowed us to analyze how to assign priorities to each of the stages of the application of process discovery. The group of clinicians always shows a high variability, which can be explained because they have fewer management tasks and their opinions vary more depending of the specific work they do [30]. Increasing the sample size of the study with new OR services from other hospitals would allow us to identify interesting preferences on clinical staff subcategories, such as surgeons, anesthesiologists, and nurses.

Another relevant finding is how the group of managers gives a high priority to the feature of comparing the process evolution over time. This finding is aligned with a previous study by Van der Aals et al., in which the feasibility of process mining tools (ProM) was analyzed in industrial contexts [31]. Technical staff assigns prioritization to the management of timestamps (date filters and occupation frequency) and information of the data cleaning process.

The quantitative prioritization of features allowed us to know the priorities each role assigned to the dashboard features. This information is of utmost importance for improving the application to provide end-users with specific tools to perform the type of analytics they can benefit the most from on a daily basis. New features (not assessed with AHP questionnaire) were also assessed in a semistructured discussion with experts. This questionnaire was based on the de facto SUS standard tool, in which we evaluated the suitability of the dashboard to fit in the daily routine of OR workers. The questionnaire was adapted, turning three closed-item questions into open-response. Respondents used these questions to indicate the possibility of creating a specific application to perform specific tasks (data filtering, process discovery, process enhancement, etc.), integrating the clinical identification of the patients instead of using the RTLS tags and using the dashboard in other contexts.

Based on the results of the quantitative and qualitative study of the features, in order to shorten the learning curve of such tools, we propose to prepare a specific dashboard interface for clinical and managerial staff, which contains only the top-ranked functionalities they have identified: Occupation maps and evolution of the processes. The remaining needed functionalities, such as data loading and filters, could be automated by the technical staff of the ORs, who were the category who assigned a higher mark to these characteristics.

Future work will focus on using the dashboard to perform advanced tasks, for instance, how errors and high noise problems (e.g., patient safety and medication issues) can be resolved using process mining as one possible application in an operating room environment.

## 6. Conclusions

The analysis of a process mining dashboard with quantitative and qualitative methodologies, such as AHP and SUS, allowed us to identify different preferences with respect to the functionalities and the role each professional has in the operating room. Despite the reduced size of the sample, our study included the complete staff of the operating rooms services of a reference hospital. Therefore, our results must be used with caution, as other findings may emerge when applying the same materials and methods in another hospital. This paper has demonstrated the ability of these techniques to identify unmet needs and user preferences when using tools for the application of process mining.

## Figures and Tables

**Table 1:** Structure and items of the filters area in the process mining dashboard under evaluation.

Item ID	Name	Area	Description
L1.1	Date	Filter	To select a specific range of dates (e.g., a day, a week, a month).
L1.2	Time	Filter	To select a specific time interval from the corpus (e.g., 9:00–14:00).
L1.3	Duration	Filter	Allows to filter traces that have a specific minimum and maximum duration (e.g., ≥ 60 min)
L1.4	Level	Filter	The real-time location system (RTLS) aggregates operating rooms (ORs) into different levels. There are levels that contain the small areas and levels that group different areas, generating more global location zones, for example, on one level, we can find “Operating Room 1”, “Operating Room 2”, etc. and on another level, these areas are grouped within the “Operating Rooms Level 0”. This filter allows users to define the granularity of the area for the process mining analysis. More than one level can be selected at the same time.
L1.5	Name of the nodes	Filter	This filter shows the names of the nodes or areas within the location of the system (e.g., Figure 1). This filter allows to indicate which areas or nodes the user wants to include in the mining (by removing the checkbox mark the node is excluded from the data corpus). This filter also allows to rename the area of location, being able to assign a new name to an area or even group two areas, giving them the same name (node aggregation).
L1.6	Features	Filter	This filter allows to select and display only the data that comply with all the characteristics that are selected (e.g., type of surgical procedure, surgeon). Such data are typically available as metadata of the RTLS.
L1.7	Stretch	Filter	This filter allows to select samples/traces which go through a given node or which go from one given source node to a given target node. It is also possible to limit the number nodes in the trace (e.g., samples with more than 3 nodes).
L1.8	Dis-gregation	Filter	This filter allows to divide the corpus of data into several corpus, grouping the samples by their percentage of similarity. Samples not matching the similarity index are grouped into an outliers pool.
L1.9	Statistics	Filter	This filters allows to display the percentage of samples of the flows that meet a given characteristic. In addition, these percentages can be grouped if they are less than a given value.

**Table 2:** Structure and items of the mining area in the process mining dashboard under evaluation.

Item ID	Name	Area	Description
L2.1	Frequency	Mining	Shows the work flow in the form of a heat map, indicating which elements occur more frequently or for a longer time.
L2.2	Occupation	Mining	Shows the work flow in the form of a heat map, indicating which nodes/locations are most occupied at a moment in time.
L2.3	Transitions	Mining	Highlights in the work flow round-trip steps (jumps) for a node.
L2.4	List of samples	Mining	Shows a list of all the samples that are in a trace or work flow. By clicking on a sample, the pathway is highlighted on the display.

**Table 3:** Structure and items of the information area in the process mining dashboard under evaluation.

Item ID	Name	Area	Description
L3.1	Sample cleaning	Information	Information related to the correction of the corpus by erasing automatically implausible samples or samples with incorrect data.
L3.2	Wrong Selection	Information	Console to display error messages in the configuration of filters, sample identification or mining.
L3.3	Evolution of process	Information	A log of actions executed when the inference engine starts (e.g., selecting data, filtering dates, filtering times, grouping samples).
L3.4	Error Messages	Information	Error messages while the inference engine is being executed (e.g., the configuration of filters entails the in-existence of samples).
L3.5	Extra information	Information	Extra information about the entire process (e.g., console messages, warnings).

**Table 4:** Questionnaire for the qualitative study of the dashboard usability.

Item ID	Question	Type of Response
Q1	I think that I would like to use the dashboard in my daily routine	5-item Likert
Q2	I found the dashboard to be simple	5-item Likert
Q3	I think the dashboard is easy to use	5-item Likert
Q4	I think I could use the dashboard without the support of a technical person	5-item Likert
Q5	I found the functions of the dashboard well integrated	5-item Likert
Q6	I thought there was a lot of consistency in the dashboard	5-item Likert
Q7	I am missing some functionalities in the dashboard	Open answer
Q8	I would remove the following functionalities from the dashboard	Open answer
Q9	I felt very confident using the dashboard and it was very intuitive	5-item Likert
Q10	I would use the dashboard in other hospital processes	Open answer

**Table 5:** Profiles of the participants in the analytic hierarchy process (AHP) study.

Variable	Type	Distribution
Role	Manager	20 %
	Clinical staff	60 %
	Technical	20 %
Age		46.2 ±10.3
Gender	Male	40%
	Female	60%
Years of expertise		21.2 ±10.7
Computer literacy	Low	0%
	Medium	70%
	High	30%

## Abbreviations

The following abbreviations are used in this manuscript:

OR	Operating room
RTLS	Real-time location system
AHP	Analytic hierarchy process
CW	Category weights
GW	Group weights
